# Self-reported glaucoma prevalence and related factors, contribution to reported visual impairment, and functional burden in a cross-sectional study in Colombia

**DOI:** 10.1007/s10792-023-02643-z

**Published:** 2023-03-02

**Authors:** Nicolás Castellanos-Perilla, Elkin Garcia-Cifuentes, Juliana Pineda-Ortega, Sofia Lema, Geronimo Gelvis, Carlos Alberto Cano-Gutierrez, Alvaro J. Mejia-Vergara

**Affiliations:** 1 Centre for Age-Related Medicine (SESAM), Stavanger University Hospital, Stavanger, Norway; 2 Department of Clinical Medicine, University of Bergen, Bergen, Norway; 3 Semillero de Neurociencias y Envejecimiento, Ageing Institute, Medical School, Pontificia Universidad Javeriana, Bogotá, Colombia; 4 Neurology Department, Hospital Universitario San Ignacio, Bogotá, Colombia; 5 Geriatrics Unit, Hospital Universitario San Ignacio, Bogotá, Colombia; 6 Department of Ophthalmology, Stein and Doheny Eye Institutes, University of California, Pasadena, Los Angeles, CA USA; 7 Ophthalmology Department, San Ignacio University Hospital, Pontificia Universidad Javeriana School of Medicine, Bogotá, Colombia; 8 School of Medicine, Ophthalmology Program, Fundación Universitaria Sanitas, Bogotá, Colombia; 9 Oftalmosanitas Eye Institute, Fundación Universitaria Sanitas, Bogotá, Colombia

**Keywords:** Glaucoma, Aged, Functionality, Fall, Quality of life

## Abstract

**Purpose:**

Describe the self-reported prevalence of glaucoma in Colombian older adults, emphasizing the most important risk factors and associated daily-life functional alterations.

**Methods:**

This a secondary analysis of the Health, Wellness, and Aging survey conducted in the year 2015. Diagnosis of glaucoma was obtained from self-report. Functional variables were assessed through activities of daily living questionnaires. A descriptive analysis followed by bivariate and multivariate regression models adjusting for confounding variables was conducted.

**Results:**

Self-reported prevalence of glaucoma was 5.67%, with higher rate in women, OR 1.22 (1.13–1.40) *p* = .003, older age OR 1.02 (1.01–1.02) *p* < .001, and with higher education OR 1.38 (1.28–1.50) *p* < .001. Glaucoma was independently associated with diabetes OR 1.37 (1.18–1.61) *p*  < .001 and hypertension 1.26 (1.08–1.46) *p* = .003. It also showed statistically significant correlations with poor SRH OR 1.15 (1.02–1.32) *p* < .001, self-reported visual impairment 1.73 (1.50–2.01) *p* < .001, and impairment in money management OR 1.59 (1.16–2.08) *p*  = .002, grocery shopping OR 1.57 (1.26–1.96) *p* < .001 and preparing meals OR 1.31 (1.06–1.63) *p * = .013 and having had falls during the last year OR 1.14 (1.01–1.31) *p* = 0.041.

**Conclusion:**

Our findings suggest the self-reported prevalence of glaucoma in older adults in Colombia to be higher than reported data. Glaucoma and visual impairment in older adults represent a public health concern, since glaucoma was associated with adverse outcomes like functional loss and risk of falling, affecting the quality of life and their participation in society.

## Introduction

Demographic transition is a worldwide phenomenon, due to a decrease in birth rates followed by a decrease in death rates. This transition is a relevant phenomenon in developing countries, increasing the number of people living with chronic illness [1].

The World Health Organization’s (WHO) Integrated Care for Older People tool package states that the visual capacity in older adults is one of the intrinsic domains to preserve [2]. Low vision is defined as visual acuity between 20/50 and 20/200 (Log Mar 0.4 to 0.9), or a visual field remanent of 20°central vision. Legal blindness is defined as visual acuity of less than 20/200 or worse, or a visual field remanent of 10° of central vision [3]. Both definitions based on the better eye with the best possible correction. Visual impairment (VI) encompasses both low vision and legal blindness [3]. In 2015, an estimated 118 million had VI in Latin America and the Caribbean; hence, it is considered a public health priority [2, 4, 5]. Although VI prevalence has declined in the past decades, older adults show a significant increase in VI with age [6–8]. VI in older adults affects quality of life (QoL), increases risk of falls and isolation with lower self-rated health (SRH) [9, 10]. VI is an independent and significant predictor of morbidity, mortality, quality of life, physical and psychological health in older adults [11].

Glaucoma is a progressive optic neuropathy characterized by specific morphological changes in the optic nerve head and nerve fibre layer of the retina [12, 13]. Glaucoma is one of the leading causes of blindness worldwide [10, 12]. Risk factors include intraocular pressure, age over 60, African or Hispanic origin, and family history. One of its major challenges is early diagnosis; half of people with glaucoma are undiagnosed in developed countries [14, 15].

The relationship between age and glaucoma has been extensively studied, with an increase in glaucoma incidence as populations live longer [16]. Aging of the trabecular meshwork leads to increased resistance and secondary aqueous humour flow reduction, and increased intraocular tension, mechanical stress in an aged lamina cribrosa, generates a pressure gradient that distorts axoplasmic transport of retinal ganglion cells [12, 17, 18]. Age may also play a role in neuroinflammation balance and in retinal environment [19].

The global prevalence of glaucoma has been estimated at 3.54%, and in Latin America at 4.51% [20]. Glaucoma contributes to around 8% of the reported blindness being the second cause of blindness in Latin America [5, 21, 22]. In Colombia, the age group with higher rates of glaucoma is those 80 and over with 1.1% estimated prevalence, followed by those 75–79 years with 1.02% [23].

Based on the need to establish the impact of glaucoma and the associated factors in older adults in Colombia, we aimed to describe its self-reported prevalence and analyse its impact in daily activities. We also explored known risk factors. For this we used data from the Health, Wellness and Aging survey 2015 survey, SABE.

## Materials and methods

### Study design

This study is a secondary analysis from the 2015 SABE study; the SABE study (from its initials in Spanish: Salud, Bienestar and Envejecimiento) comprised 23,694 older adults, non-institutionalized adults aged 60 years and older. Initially conducted by the Pan-American Health Organization (PAHO) and conducted in Colombia in 2015 with a multistage area probability sampling design, supported by a fund from Colombian Science Ministry and the Colombian Ministry of Health and Social Protection Data collection was carried between April and September 2015 with 62% urban and 77% rural response rates [24].

### Variables

Most variables were obtained through self-report. The dependent variable was the self-report of history of glaucoma (assessed with the question: “Has a doctor ever told you to require treatment for glaucoma?”, Yes/No).

Vision impairment was evaluated by self-report as well (with the question “How would you describe your vision? Possible answers included: Very good, good, poor, bad and very bad; we dichotomized answers into bad (very bad, bad, and poor) and good (good–very good)).

The questions addressing performance on instrumental activities of daily living (IADL) and need of help were evaluated for the VI report. These questions have been detailed in the survey’s complete method [24].

#### Confounding variables

We included the sociodemographic factors: sex, age, educational level measured with years of schooling, and cognitive decline evaluated with the Mini-Mental State Examination (a score < 24 was the cutoff value for defining cognitive impairment). We included multi-morbidity, defined as reporting ≥ 2 chronic diseases including hypertension (HTN), diabetes (DM), chronic obstructive pulmonary disease (COPD), stroke, myocardial infarction, arthritis/osteoporosis, and cancer.

#### Exclusion criteria

**Fig. 1:**
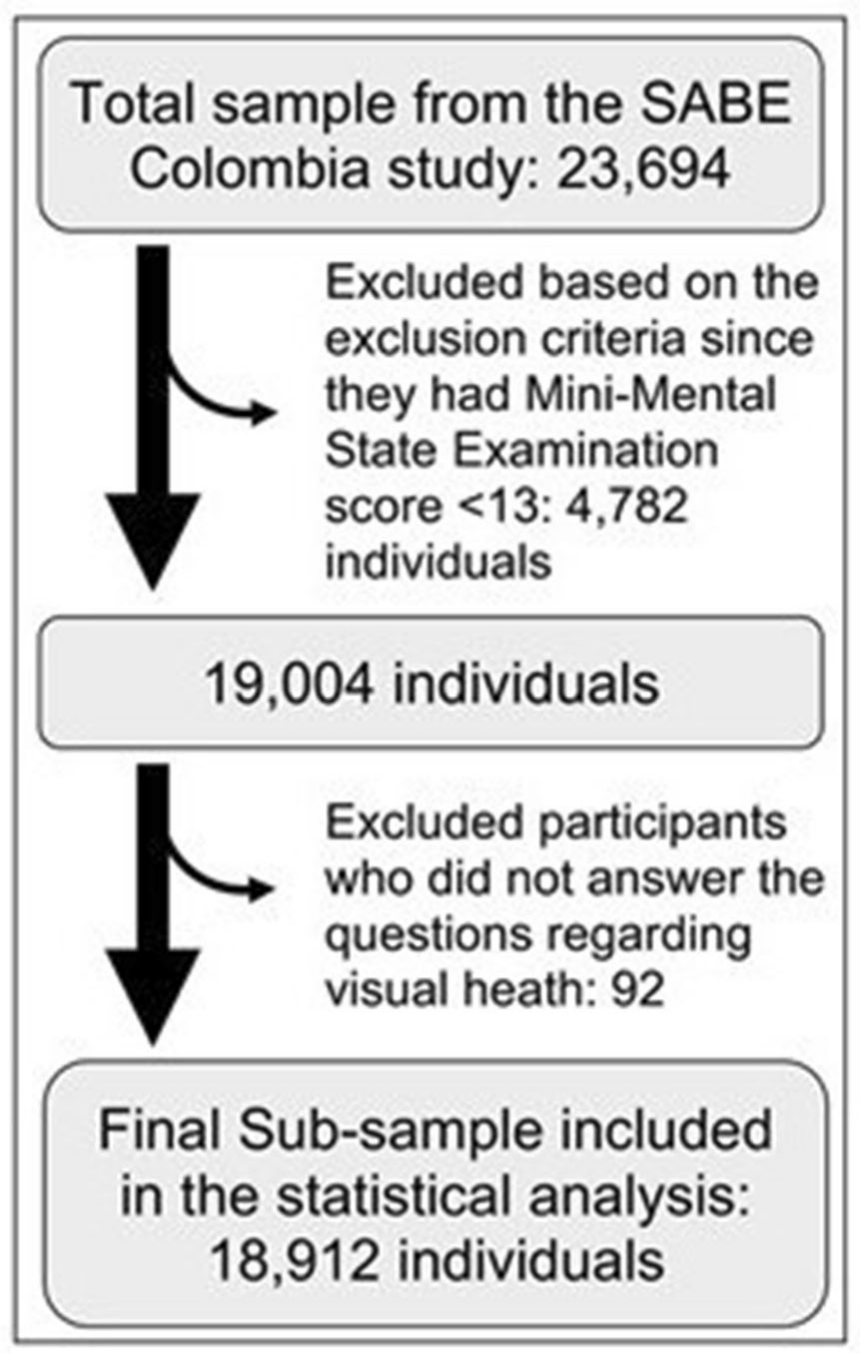
Exclusion criteria applied on SABE Colombia study

We excluded participants who did not answer the questions regarding visual heath. (Fig. 1) Individuals were considered unable to complete the study procedures and excluded at the beginning of the interview if they had a total score of < 13 in the Mini-Mental State Examination (MMSE), and a proxy interview was developed.

### Statistical analysis

A descriptive analysis was done using relative and absolute frequencies for nominal variables, and median and interquartile range (IQR) were used for continuous variables. A Shapiro–Wilk test was used for testing normality.

For the bivariate analysis, statistical significance was assessed using the chi-squared test for categorical variables and the ANOVA tests for continuous variables.

Logistic regression models were fitted to obtain odds ratios (OR) with 95% confidence intervals (CI). An additional linear regression model was built adjusting for the confounding variables. Statistical significance level was set at p < 0.05. Data were analysed using STATA 16 (Stata Corp. LLC Texas, USA).

## Results

The total surveyed sample was 23,694 individuals. We excluded 4782 subjects (17%) based on the exclusion criteria. (Fig. 1).

Out of the 18,912, 5.67% had glaucoma. The majority were female in both groups, with a higher proportion in the glaucoma group 62.03% vs 55.71% (*p* < 0.001). Median age was 71 years (SD ± 8.26) in the glaucoma group vs 69.0 years (SD ± 8.15) in the no-glaucoma group (*p* < 0.001). Mean education years were 4 years in the glaucoma group vs3 years in people without glaucoma (*p* < 0.001). In the glaucoma group, 26.8% had DM diagnosis and 65.73% HTN vs v 15.78% (*p* < 0.001) and 51.30% (*p* < 0.001) in the no-glaucoma group. Multi-morbidity proportion was more prevalent in the subjects with glaucoma when compared to those without, 58.86% vs 39.20% (*p* < 0.001). (Table 1).

**Table 1:** Bivariate analysis

Glaucoma	Yes	No	
	n = 1072 (5.67%)	n = 17,840 (94.33%)	*p*
	*Median (IQR)*X		
Age	71 (± 8.26)	69 (± 8.15)	< 001
Years in school	5 IQR (5)	3 IQR (4)	< .001
	*n (%)*		
Females	665 (62.03)	9939 (55.71)	< .001
Diabetes	287 (26.8)	2805 (15.78)	< .001
Hypertension	704 (65.73)	9140 (51.30)	< .001
Poor self-rated health	628 (58.64)	9192 (51.53)	< .001
Multi-morbidity	631 (58.86)	6994 (39.20)	< .001
Vision impairment	826 (77.05)	11,565 (64.83)	< .001
Cognitive decline	108 (10.6)	2286 (12.81)	.324
Fear of falling	903 (84.39)	14,250 (80.02)	< .001
Falls in the previous year	379 (35.35)	5231 (29.22)	< .001
Difficulty for IADL	300 (27.99)	4748 (26.61)	.321
Difficulty managing money	54 (5.04)	479 (2.68)	< .001
Difficulty shopping for groceries	108 (10.07)	942 (5.28)	< .001
Difficulty preparing meals	111 (10.35)	1341 (7.52)	< .001
Difficulty taking transportation	164 (15.30)	2142 (12.01)	< .001
Difficulty using the telephone	164 (15.30)	2142 (12.01)	< .001
Does not read	250 (26.12)	3747 (25.33)	.585
Does not write	657 (68.72)	10,265 (69.45)	.638
Difficulty taking medications	71 (6.62)	1023 (5.73)	.226
Depressive symptoms	372 (34.70)	6520 (36.55)	.223

*SD* standard deviation, *IQR* interquartile range, *IADL* instrumental activities of daily living

The self-reported VI rate was higher in the group with glaucoma with of 77.05% compared to 64.83% (p < 0.001) in the group without glaucoma. We found a significant difference between the group with and the one without glaucoma in fear of falling 84.39% vs 80.02% (*p* < 0.001). (Table 1).

Having had falls in the previous year was more frequently reported in the glaucoma group, when compared to the non-glaucoma group. 35.35% vs 29.22% (*p* < 0.001) poor SRH 58.64 vs 51.52% (*p* < 0.001) was also more frequently reported in that group. Analysing impairment in IADL, we found difficulties in the following: managing money 5.04% (glaucoma) vs 2.68% (without glaucoma) (*p* < 0.001), shopping for groceries 10.07% (glaucoma) vs 5.28% (without glaucoma) (*p* < 0.001), cooking 10.35% vs 7,52% (*p* < 0.001), use of transportation 15.30% vs 12.01% (*p* < 0.001), and using the telephone 15.30% vs 12.01% (*p* < 0.001). (Table 1).

**Fig. 2:**
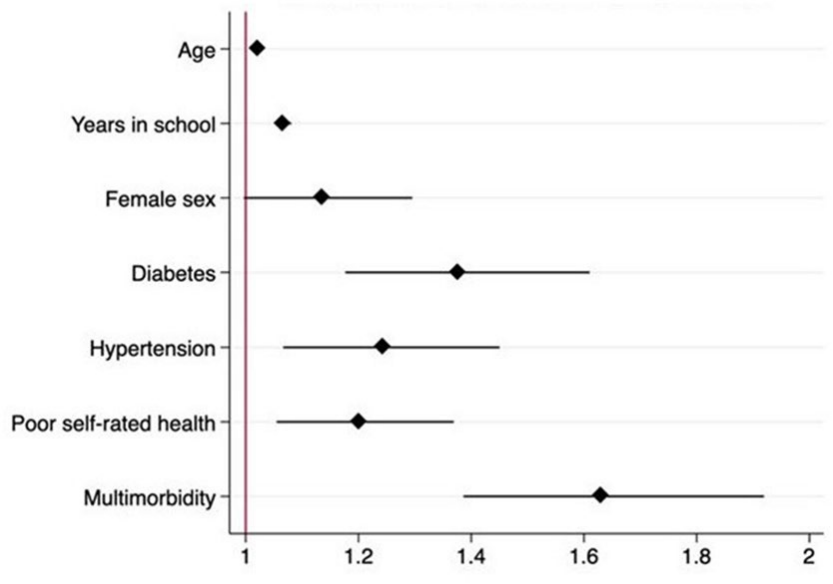
Multivariate analysis of Glaucoma and related risk factors. OR odds ratio (x axis). (95%CI). Adjusted by confounding factors

**Fig. 3:**
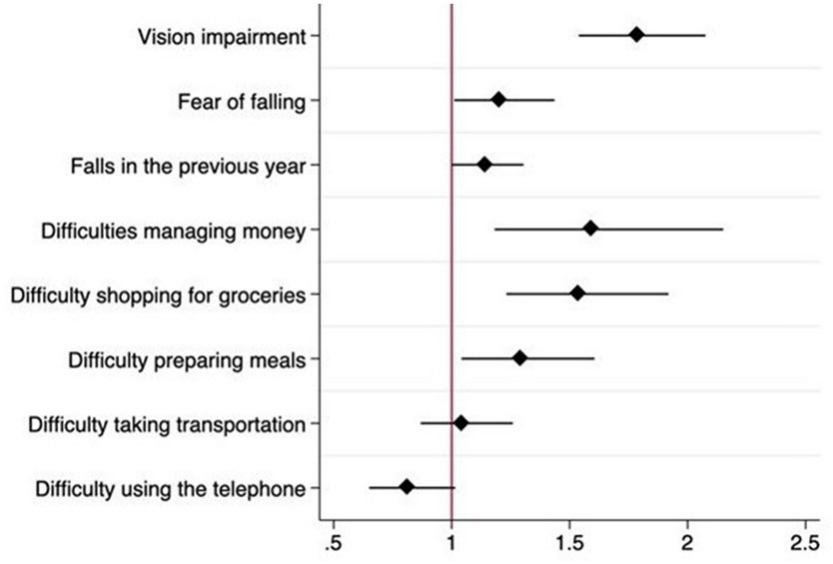
Multivariate analysis of Glaucoma and adverse outcomes. OR odds ratio (x axis). (95%CI). Adjusted by confounding factors

The multivariate analysis shows the following variables were independently associated with the self-report of glaucoma, after adjusting for confounding variables (Figs. 2 and 3): female sex OR 1.22 (1.13–1.40) *p* = 0.003 older age OR 1.02 (CI 1.01–1.02), *p* < 0.001, higher school level OR 1.36 (CI 1.28–1.50), *p* < 0.001, DM OR 1.37 (CI 1.18–1.61), *p* < 0.001, HTN OR 1.26 (CI 1.08–1.46), *p* < 0.003, and multi-morbidity OR 2.08 (CI 1.83–2.37), *p* < 0.001. (Table 2).

**Table 2:** Multivariate analysis

	OR (SD)	*p*	Adjusted OR	*p*
Age	1.02 (1.01–1.02)	< .001	1.02 (1.01–1.02)	< .001
Years in school	1.36 (1.26–1.46)	< .001	1,06 (1.05–1.07)	< .001
Females	1.26 (1.13–1.42)	< .001	1.22 (1.13–1.40)	.003
Diabetes	1.95 (1.69–2.25)	< .001	1.37 (1.18–1.61)	< .001
Hypertension	1.82 (1.59–2.07)	< .001	1.26 (1.08–1.46)	.003
Poor self-rated health	1.33 (1.17–1.51)	< .001	1.15 (1.02–1.32)	.027
Multi-morbidity	2.21 (1.95–2.51)	< .001	2.08 (1.83–2.37)	< .001
Vision impairment	1.82 (1.57–2.10)	< .001	1.73 (1.50–2.01)	< .001
Fear of falling	1.35 (1.14–1.59)	< .001	1.20 (1.01–1.43)	.038
Falls in the previous year	1.31 (1.16–1.47)	< .001	1.14 (1.01–1.31)	.050
Difficulties managing money	1.92 (1.44–2.56)	< .001	1.59 (1.16–2.08)	.002
Difficulty shopping for groceries	2.01 (1.62–2.48)	< .001	1.57 (1.26–1.96)	< .001
Difficulty preparing meals	1.42 (1.15–1.74)	< .001	1.31 (1.06–1.63)	.013
Difficulty taking transportation	1.32 (1.11–1.57)	< .001	1.06 (0.88–1.27)	.537
Difficulty using the telephone	0.78 (0.63–0.97)	.027	0.83 (0.66–1.03)	.101

*Adjusted by sex, age, years of school, multi−morbidity, cognitive decline, and hearing impairment. *OR* odds ratio, *SD* standard deviation, *f* female

Presence of glaucoma was significantly related to VI OR 1.73 (1.50–2.01), *p* < 0.001, impairment in instrumental activities in managing money OR 1.59 (1.16–2.08) *p* = 0.002, shopping for groceries OR 1.57 (CI 1.26–1.96) *p* < 0.001 and preparing meals OR 1.31 (1.06–1.63), *p* = 0.013; having had falls during the last year OR 1.14 (CI 1.01–1.31), *p* = 0.050, poor SRH OR 1.15 (CI 1.02–1.32), *p* = 0.027. (Table 2).

## Discussion

The relationship between glaucoma and age has been well described [12, 25, 26]. Our findings are the first to expose the extend of the association in Colombia. The glaucoma group had a mean age 2 years higher. This could account for higher comorbidity and higher glaucoma risk, however when adjusting for multi-morbidity and other confounding factors, age remained statistically significant related to glaucoma report.

As reported by other groups, our cohort had a higher glaucoma rate among women [6]. This might be explained by women’s increased longevity. Other series have failed to demonstrate a real gender predilection after statistical controls [27]. Although low oestrogen levels have been reported as a risk factor for optic nerve changes, its role in glaucoma is yet to be completely understood [28, 29].

Both HTN and DM had a significant association with glaucoma in our study. Research has showed up to 35% increased risk of developing open angle glaucoma in subjects with DM and 17% in HTN [27, 30]. Although DM and HTN are prevalent in old age, our data supports the notion that both entities have an independent effect on glaucoma report. Several authors have speculated that changes in systemic blood pressure may contribute to dysregulation of the intraocular pressure [31, 32].

Sociodemographic factors appear to have a significant impact in glaucoma. People with self-report of glaucoma diagnosis have higher educational levels in our sample. Higher education increases health access opportunities, allowing subjects to larger health access with regular eye examinations, allowing for earlier diagnosis [33, 34]. Education is also important as an intervention for treatment adherence [35].

Glaucoma is one of the major causes of irreversible vision loss worldwide [20], with a large impact on QoL [10], we found subjects with glaucoma to be more likely to have poor SRH. Glaucoma generates a significant VI with impairment for carrying out daily tasks such as walking outside, reading, seeing at night, adapting to lightning, and tasks that need discrimination of depths and distances [36–38]. This has been related to poor performance in different visual tasks (contrast sensitivity, dark adaptation, glare disability, and visual field tests) [39]. Based on this, VI has a negative impact on physical and mental health [36]. Previous reports have shown an increased risk of falling and having accidents, social isolation, and depression in glaucoma patients [40, 41]. Our study did not show that the self-report of glaucoma associated with depressive symptoms. We also found glaucoma to be related to fear of falling, after adjusting for falls during the previous year, as reported by other groups [42].

Managing money, shopping for groceries, and preparing meals were also affected in the glaucoma group. Previous research has shown evidence for decline in activities of daily living, and decreased QoL in glaucoma patients compared with controls, even at early stages, QoL decreases as glaucoma severity advances [43–46]. Vision loss in glaucoma can affect activities such as walking, venturing out from home, reading, seeing at night, adjusting to different levels of illumination, judging distances, driving, and seeing objects coming from the side with difficulties with central, near vision tasks, and reading the most often compromised [36, 37, 39].

Glaucoma is a condition of great concern that requires strategies for early detection, still underdiagnosed, with social factors like education and health access determining adequate diagnosis that in the long-term can improve functionality in the older adults, reduce risk of falling, overall morbidity, and mortality, and improve QoL for older adults. Latin America is living longer, and age is one of the most important risk factors for Glaucoma [30]. Our findings show a higher self-reported prevalence of glaucoma in the Colombian population, than earlier reported rates [23]. This may be explained by the older age of the cohort.

The present study presents several limitations. The cross-sectional survey design does not allow to confirm causality between the studied factors and glaucoma. We did not have access to medical records that could allow for verifying reports. Likewise, most of the variables are self-reported, which may lead to numbers different to the actual ones, as is the diagnosis of glaucoma. To address these biases, we limited our data to those participants without cognitive decline [24]. Other studies highlight participants may underestimate or overestimate their conditions and visual abilities [47]. In this sense, self-report may be influenced by the frequency the person assists to doctor visits. This may not only affect the reported prevalence of glaucoma, but also other conditions, and be associated to the positive relation with socioeconomic status and health access. However, we used a significant and representative sample and adjusted for possible confounding variables and found various significant associations that may contribute to the approach of the disease in the clinical and public health context.

Although prevalence estimates have been given in Latin America and Colombia, it is important to further explore factors related to glaucoma in other age groups. With glaucoma being a preventable and treatable disease with serious outcomes, our study is one of the few exploring the self-reported prevalence and associated factors to glaucoma in Colombian older adults. Appropriate glaucoma diagnosis must be followed by an adequate treatment with the goal of delaying disease progression and providing long-term visual function and QoL at a reasonable cost, compliance to treatment will be paramount to prevent vision loss [48].

We show modifiable risks factors for glaucoma that need to be addressed to improve overall older adult health, aiming for better functional performance and QoL in those 60 and over, this paper reflects the need of research around visual health in Latin America hoping to improve medical attention and prevention of glaucoma.
